# Statistical Methods Used to Test for Agreement of Medical Instruments Measuring Continuous Variables in Method Comparison Studies: A Systematic Review

**DOI:** 10.1371/journal.pone.0037908

**Published:** 2012-05-25

**Authors:** Rafdzah Zaki, Awang Bulgiba, Roshidi Ismail, Noor Azina Ismail

**Affiliations:** 1 Julius Centre University of Malaya, Department of Social and Preventive Medicine, Faculty of Medicine, University of Malaya, Kuala Lumpur, Malaysia; 2 Department of Applied Statistics, Faculty of Economics and Administration, University of Malaya, Kuala Lumpur, Malaysia; University of East Piedmont, Italy

## Abstract

**Background:**

Accurate values are a must in medicine. An important parameter in determining the quality of a medical instrument is agreement with a gold standard. Various statistical methods have been used to test for agreement. Some of these methods have been shown to be inappropriate. This can result in misleading conclusions about the validity of an instrument. The Bland-Altman method is the most popular method judging by the many citations of the article proposing this method. However, the number of citations does not necessarily mean that this method has been applied in agreement research. No previous study has been conducted to look into this. This is the first systematic review to identify statistical methods used to test for agreement of medical instruments. The proportion of various statistical methods found in this review will also reflect the proportion of medical instruments that have been validated using those particular methods in current clinical practice.

**Methodology/Findings:**

Five electronic databases were searched between 2007 and 2009 to look for agreement studies. A total of 3,260 titles were initially identified. Only 412 titles were potentially related, and finally 210 fitted the inclusion criteria. The Bland-Altman method is the most popular method with 178 (85%) studies having used this method, followed by the correlation coefficient (27%) and means comparison (18%). Some of the inappropriate methods highlighted by Altman and Bland since the 1980s are still in use.

**Conclusions:**

This study finds that the Bland-Altman method is the most popular method used in agreement research. There are still inappropriate applications of statistical methods in some studies. It is important for a clinician or medical researcher to be aware of this issue because misleading conclusions from inappropriate analyses will jeopardize the quality of the evidence, which in turn will influence quality of care given to patients in the future.

## Introduction

Most important variables in medicine are measured in numerical forms or continuous data, such as blood pressure, glucose level and oxygen level. In any clinical situation, we are expected to have accurate readings of these variables. Numerous new techniques or tools have been developed with the aim of finding a cheaper, non-invasive, more convenient and safer method to test patients. It is important to be sure that the new tool or method of measurement is as accurate as the current or gold standard method. Therefore it is important to measure the agreement of the new method with the standard method. Agreement signifies the accuracy of that certain instrument [1].

Various statistical methods have been used to test for agreement of medical instruments with quantitative or continuous outcomes [2], [3]. Which method is the best is still open to debate and almost all methods have been criticized. The old favorite for measuring agreement is the correlation coefficient (r) [4]. However, this is obviously inappropriate as correlation only measures the strength of linear association between variables. Coefficient of determination (r^2^), regression coefficient, and comparing means have also been shown to be inappropriate ways of assessing agreement. This was discussed by Altman and Bland in their article [2] back in the 1980s. Their conclusions on the inappropriate methods to assess agreement have been supported by Daly and Bourke [4], and there is little argument about this in the literature.

Bland and Altman proposed a method for the analysis of agreement (Bland-Altman plot and limits of agreement) in 1983 [2] and later drew the attention of the medical profession to this area in their article [5] in the Lancet. They stated that it is very unlikely for two different methods or instrument to be exactly in agreement, or to give identical results for all individuals [5]. What is important is how close the pairs of values are [5]. This is because a very small difference in the predicted and the actual value is not likely to affect patient management decisions [5]. Since then their article received a large number of citations in the literature. This leads one to think that the Bland-Altman method is the most popular statistical method used in agreement studies and that other methods are no longer being used in this kind of research as almost 30 years have passed since the original article first appeared promoting the new method. However, citation does not imply that this method has been applied in research. Comments or critiques of the method could also contribute to the high number of citations of any article. There is no previous systematic review in the literature to establish whether the Bland-Altman method is indeed the most popular method used in agreement studies.

The purpose of this study is to review statistical methods used to assess agreement of medical instruments measuring the same continuous variable in the medical literature. The proportion of various statistical methods found in this review will also reflect the proportion of medical instruments that have been validated using those particular statistical methods in current clinical practice.

## Methods

This review follows the reporting standards as suggested in the PRISMA statement; see PRISMA Checklist S1.

### Searching

#### Eligibility criteria

Any method comparison studies assessing the agreement of medical instruments or equipment. Only the agreement of continuous variables will be considered. The instruments must be applicable for use in humans.

#### Search strategy for identification of studies

In 2010, we searched Medline, Ovid, PubMed, Scopus and Science Direct for studies investigating the agreement of instruments or equipments in medicine published in journals between January 2007 and December 2009. Boolean search was performed on each database using the search term: Agreement AND (validation OR “comparison study”). The search was limited to the medical field (including dentistry), studies involving human subjects, and articles in the English language.

### Study Selection

All citations identified from the search were downloaded into the EndNote X1 software. The citations were organized and duplicates were identified and deleted. We excluded any studies with qualitative or categorical data, studies with different units of outcomes, and association studies. Unpublished articles were not considered in this review. Study selection was conducted by two independent researchers. There was no disagreement between the two reviewers at the stage of study selection.

### Data Extraction

We extracted characteristics from each article based on the year of publication and journal type. We categorized journal types into five areas: medicine (including obstetrics, gynecology, emergency and critical care medicine), surgery, radiology, nutrition and others.

We collected information on the statistical methods used to assess agreement from the methodology section or the statistical analysis section, and also by identifying which statistical methods influenced the author’s conclusion on the agreement.

Data extraction was performed by two researchers independently. Most of the time, the two researchers agreed with the outcomes. In any case of disagreement, agreement was reached by consensus, and a third reviewer assisted when consensus could not be reached.

Descriptive analysis of the characteristics of studies and statistical methods used were performed. This is a descriptive review, and all results are displayed as percentages. Data were analyzed using the SPSS 15.0 software.

## Results

**Figure 1:**
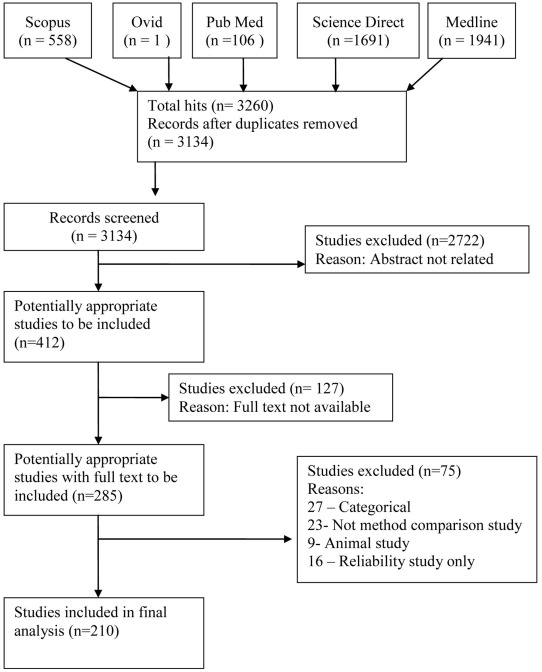
Flowchart of studies.

A total of 3,260 titles were initially identified, and after filtering for duplicates 3,134 records (titles and abstracts) were screened. Only 412 titles were potentially related and 285 full-text report records were reviewed. Seventy-five articles did not meet the inclusion criteria, and a total of 210 articles were finally included in this review. Figure 1 summarizes the selection process.

Out of the 210 articles reviewed, 70 were published in 2007, 70 in 2008, and 70 in 2009. Eighty-eight (42%) of the articles were obtained from the Science Direct database, 51 (24%) from the Medline database, 48 (23%) from the Scopus database, and 23 (11%) from the PubMed database. Most of the studies (72 or 34%) were published in medical journals, 30 (14%) in nutrition-related journals, 29 (14%) in radiology journals, and 28 (13%) in surgical journals.

Overall, 117 articles (56%) used a single method to assess agreement while 93 articles (44%) used multiple (two or more) methods. The most popular statistical methods used to assess agreement in the 210 reviewed articles and according to specialty are summarized in Table 1 and Table 2. Most of the articles (178 articles or 85%) used the Bland-Altman method (Limits of Agreement) to measure the agreement of equipment. Out of the 178 articles, 99 (56%) used the Bland-Altman method alone to assess agreement while the remainder (79 or 44%) combined the Bland-Altman method with another method.

**Table 1:** Most popular statistical methods used to assess agreement in medicine.

Statistical Method Used	Number of articles using the method, x (%) n = 210
1. Bland-Altman Limits of Agreement2. Correlation coefficient (r)3. Compare means/Significant test4. Intra-class Correlation Coefficient5. Compare slopes or/and intercepts	178 (85%)58 (28%)38 (18%)14 (7%)13 (6%)

n = Total number of studies retrieved, x =  number of studies, %  =  percentage.

**Table 2:** Most popular statistical methods used to assess agreement according to area of specialty in medicine.

Area of specialty	Statistical Method Used	Number of articles using the method (x)
**Medicine (n = 29)**	1. Bland-Altman Limits of Agreement2. Correlation coefficient (r)3. Compare slopes or/and intercepts4. Intra-class Correlation Coefficient5. Compare means/Significant test	246432
**Surgery (n = 25)**	1. Bland-Altman Limits of Agreement2. Correlation coefficient (r)3. Compare means/Significant test4. Intra-class Correlation Coefficient5. Percentage of error	218541
**Radiology (n = 29)**	1. Bland-Altman Limits of Agreement2. Correlation coefficient (r)3. Compare means/Significant test4. Intra-class Correlation Coefficient5. Compare slopes/intercepts	266632
**Nutrition (n = 30)**	1. Bland-Altman Limits of Agreement2. Correlation coefficient (r)3. Coefficient of determination (r^2^)4. Compare means/Significant test5. Compare slopes or/and intercepts	2513444

n = Total number of studies retrieved for each specialty, x =  number of studies.

Twenty articles [6], [7], [8], [9], [10], [11], [12], [13], [14], [15], [16], [17], [18], [19], [20], [21], [22], [23], [24], [25] used either the correlation coefficient, coefficient of determination, comparison of means, or a combination of these methods in the analysis of agreement. Table 3 shows some of the examples of inappropriate applications and interpretations of statistical analysis in the analysis of agreement found in this review.

**Table 3:** Examples of inappropriate applications and interpretations of statistical analyses to assess agreement found in this review.

	Study objective	Results & author’s conclusion
Ten 2007 [8] n = 355	To compare four different commercial activated partial thromboplastin time (aPTT) reagents to detect shortened aPTT.	Correlation coefficients among the four methods ranged from 0.51 to 0.83 (all P values <0.001). Acceptable agreement between the different commercial reagents was found with respect to detection of short aPTT. Good agreement were found between Instrumentation Laboratory and bioMerieux reagents (r = 0.74–0.83)
Reis 2007 [7] n = 30	To validate a method for the quantification ofthe very low levels of urinary human chorionic gonadotropin (hCG).	Equation from regression analysis: y = 0.99x+8.55, Correlation coefficient of 0.993 demonstrates very good immunoassay accuracy for the studied range of hCG concentrations.
Satia 2007 [6] n = 658	To assess the degree of agreement betweenthree instruments of measuring dietaryfat consumption.	Pearson’s correlation coefficients among the three methods ranged from 0.18 to 0.58(all P values <0.0001). There was good concordance among the three methods.
Mündermann 2008 [14] n = 62	To compare three dimensional position capture with skin markers and radiographicmeasurement for measuringmechanical axis alignment.	The mechanical axis alignment from position capture correlated well with thegold standard of measurement using radiographs (R^2^ = 0.544 P<0.001). Theproposed method allows the measurement of the mechanical axis alignmentwithout exposure to radiation.
Anderst 2009 [18] n = 17	To compare the bead-based method oftracking bone motion in vivo with themodel-based method.	Agreement between the two systems was quantified by comparing bias (mean difference). All bias measures not significantly different from zero. The newmodel-based tracking achieves excellent accuracy without the necessityfor invasive bead implantation.
Naidu 2009 [22] n = 95	To evaluate the validity of the Hand Assessment Tool (HAT) and Disabilities of Arm, Shoulder,and Hand Questionnaire (DASH).	Strong positive correlation between DASH and HAT (r = 0.91). The HAT mayserve as useful alternative to the DASH.

## Discussion

Our study is the first systematic review on this topic. This study provides evidence that the Bland-Altman method (limits of agreement) is the most popular method that has been used to measure agreement. The majority (85%) of agreement studies in this review have applied the Bland-Altman method to assess agreement, with more than half (56%) of them using only the Bland-Altman method (i.e. without any combination with other method). Our study shows that there still inappropriate applications of statistical methods to assess agreement in the medical literature.

Bland and Altman introduced the limits of agreement to quantify agreement way back in 1983 [2]. The formula for the limits of agreement is given as: Limits of Agreement  =  mean difference ±1.96 x (standard deviation of differences). The limits of agreement is dependent on the assumptions that the mean and standard deviation of the differences are constant throughout the range of measurement, and that the distribution of these differences approximately follows a normal distribution [2]. Bland and Altman proposed a scatter plot of the difference of two measurements against the average of the two measurements and a histogram of the differences to check these assumption [2]. The scatter plot was initially used only to check these assumptions and not for the analysis of agreement, but it has since become a graphical presentation of agreement. This plot is actually similar to the Tukey mean-difference plot [26], which is popularly used in non-medical fields. This plot was popularized by Altman and Bland in medical statistics, and is now referred to as the Bland-Altman plot [26]. Despite the popularity of the Bland-Altman method, Hopkins [27] demonstrated that the Bland-Altman plot tends to incorrectly indicate the presence of systematic bias in the relationship between two measures. If a regression line was fitted to the Bland-Altman plot, it was argued that proportional bias existed if the gradient of the slope significantly differed from zero [28]. However, Ludbrook [28] claimed that the presence of bias in the analysis was due to some kind of statistical assumption. An approach using least-products regression to fit the regression line in the Bland-Altman plot has been claimed to eliminate the bias problem in the Bland-Altman plots [28].

In this review, the correlation coefficient was also found to be a statistical method used to measure agreement. Correlation Coefficient (r) reflects the noises and direction of linear relationship [4]. Perfect correlation occurs if all the points lie along a straight line. If we compare two instruments (A and B) with variable Y as the reading from instrument A and X as the reading from instrument B, it is possible to have perfect correlation (r  = 1) for both situations of Y = X or Y = 2X. However in terms of agreement, we can say that there is an agreement in the first case of Y = X, but not for Y = 2X. It is obvious that the value of Y is twice the value of X (i.e. no agreement). Table 4 demonstrates this. The correlation coefficient r for the relationship between variables A and B is 0.9798. Although the variable C is twice the value of B, the correlation coefficient of A and C is exactly the same (r = 0.9798). It is obvious that there is no agreement between A and C, but the correlation coefficient value is still very high suggesting a strong correlation or association. Therefore, it is clear that the correlation coefficient does not represent agreement.

**Table 4:** Data sets to demonstrate the inappropriate use of correlation coefficient in testing agreement.

Reading	A	B	C (twice of B)
12345678910	10.208.208.709.609.608.209.407.006.6010.80	10.208.008.059.709.058.158.806.556.5510.50	20.4016.0016.1019.4018.1016.3017.6013.1013.1021.00

Some people proceed to regression analysis as an extension to correlation analysis to answer their question of agreement. They use the coefficient of determination (r^2^) as a measure of agreement. Again, this is inappropriate because coefficient of determination (r^2^), being related to the correlation coefficient relies on a similar concept and is thus not suitable for assessing agreement. Coefficient of determination (r^2^) is used to state the proportion of variance in the dependent variables that is explained by the regression equation or model [4]. The more closely the points in the scatter diagram are dispersed around the regression line, the higher the proportion of variation will be explained by the regression line, thus the greater the value of r^2^ [4].

The third most popular method found in this review is comparing means of readings from two instruments. Paired t-test is usually used to test the significant differences between the means of two sets of data, to assess the agreement [2]. People have interpreted that non-significant results mean no differences, thus there is an agreement between the two groups and vice versa. However, the paired t-test with non-significant result does not indicate agreement. The reason for this is that the value of mean is affected by the value of each data, which leads to undue influence by extremely large or extremely small values. It is possible that poor agreement between the two instruments can be hidden in the distribution of differences, and thus the two methods can appear to agree. In an agreement study, we are not interested in the mean of readings by each instrument but we are interested in each individual reading. What matters is that each reading from the standard instrument should be repeated by the new instrument. Furthermore, significance is related to the power of the study.

Another method that was used to assess agreement found in this review is the intra-class correlation coefficient. The intra-class correlation coefficient (ICC) was initially devised to assess reliability [29]. However, it was then used to assess agreement to avoid the problem of linear relationship being mistaken for agreement in product moment correlation coefficient (r) [30], [31]. Different assignments of measurements of X and Y in the calculation of the correlation coefficient (r), would produce different values of r. To overcome some of the limitations of the correlation coefficient (r), the ICC averages the correlations among all possible ordering of the pairs [32]. The ICC also extends to more than two observations in contrast with the correlation coefficient (r). A number of different ICC statistics have been proposed, and there has been considerable debate about which ICC statistic is appropriate to assess agreement (30). The use of ICC in assessing agreement has also been criticized by Bland and Altman [31]. The ICC ignores ordering and treats both methods as a random sample from a population of methods [31]. In an agreement study, there are two specific methods that will be compared, not two instruments chosen at random from some population. Another issue with ICC is that it is influenced by the range of data. If the variance between subjects is high, the value of ICC will certainly appear to be high [33]. Although the use of the ICC seems to be popular, the appropriateness of this method to assess agreement is also doubtful.

Often in testing for agreement, the gradient of the regression line of two variables is tested against one [2]. The argument was that if the two methods or instruments were equivalent i.e. if it measures the same variable of the same subject both instruments will give the same reading, thus the gradient of the regression line would be one [2]. So if instrument A measures ‘y’, and instrument B measures ‘x’, and if y = x, the gradient of the slope is equal to one. It is true that the regression line of y = x will always have gradient  = 1. However this is not always true reversely. If the gradient  = 1, the regression line could be y = x, or could be y = a +x. Therefore, solely testing the gradient  = 1 is also an inappropriate method of testing agreement. When the gradient = 1, some people proceed to test the y-intercept. Theoretically, if gradient  = 1 and y-intercept  = 0, then y will be equal to x (y = x). However testing both gradient and intercept to assess agreement is not so popular compared to other methods.

The proportion of various statistical methods found in this review probably reflects the proportion of medical instruments that have been validated using those particular statistical methods in current clinical practice. Almost all methods have received criticism, including the Bland-Altman method. However, correlation coefficient, coefficient of determination, regression coefficient, and comparing means are obviously inappropriate to assess agreement. Although Altman and Bland have been highlighting the issue of inappropriateness of these statistical methods in method comparison studies since the 1980s, some of these methods were still in use in the studies which we reviewed. This study found that 20 (10%) of reviewed articles have used only these inappropriate methods to assess agreement. The equipment which has been tested using these methods may not be valid, and consequently may produce inaccurate readings. It makes uncomfortable reading that as many as one out of ten supposedly validated instruments currently used in clinical practice may not be accurate. This has the potential to affect the management of patients, quality of care given to the patient, and worse still could cost lives.

In 2009, Essack et al. [34] conducted a study to assess the accuracy and precision of five currently available blood glucose meters in South Africa. The study compared five different types of glucometers and all the glucometers were calibrated [34]. The authors found that although all the devices showed satisfactory precision, there was substantial discordance when their results were compared to a laboratory reference [34]. Only three out of the five glucometers fulfilled the criteria suggested by the International Standardization Organization [20]. The variability observed with the accuracy of glucometers can impact patient care in different settings, some of which include the diabetic patient on insulin in a home care or in a clinic setting. Inaccuracies can lead to misclassification of hypoglycaemic or hyperglycaemic episodes.

It is imperative that all medical instruments are accurate and precise. Otherwise, a failure in this regard may lead to critical medical errors. Therefore there is a necessity for proper evaluations of all medical instruments, and it is important to be sure that the appropriate statistical method has been used. The inappropriate application of statistical methods in the analysis of agreement is cause for concern in the medical field and cannot be ignored. It is important for medical researchers and clinicians from all specialties to be aware of this issue because inappropriate statistical analyses will lead to inappropriate conclusions, thus jeopardizing the quality of the evidence, which may in turn influence the quality of care given to the patient.

Of the 210 reviewed articles, only six studies were co-authored by someone working in a statistics or biostatistics department. Other studies did not state whether any assistance was sought from a statistician. One of the six studies have used correlation coefficient and comparing means to study agreement, whereas the other five studies have used the Bland-Altman method (either singly or in combination with another method). Medical researchers might need to consider assistance from a statistician in analyzing data from agreement studies. This could potentially reduce errors in data analysis, avoiding the use of inappropriate methods and improve the interpretation of results in their studies.

Recently, the guidelines for reporting reliability and agreement studies (GRRAS) have been proposed [35]. Kottner et al. [35] found that reporting of method comparison studies (both agreement and reliability studies) were incomplete and inadequate. Information about sample selection, study design and statistical analysis were often incomplete [35]. We also found that even a recent article [36] published early this year in 2012, relied on inappropriate analysis to test for agreement. The authors in this article [36] have used the r-squared (r^2^) or also known as the coefficient of determination to assess the accuracy (agreement) of glucose analyzers. In one of their results, the authors described that the Nova StatStrip device showed excellent performance that almost agreed and correlated perfectly with the lab results because the r^2^ = 0.99 [36]. This suggests that there is a need for a recommendation or guideline on how to perform analysis in agreement studies.

This systematic review has several strengths. This is the first study specifically designed to retrieve information on statistical methods used to test for agreement of instruments measuring the same continuous variable in the medical literature. This study also provides supporting evidence that confirms the anecdotal claim that the Bland-Altman method is the most popular method used to assess agreement. A broad search term was used, in order to capture the largest possible number of publications on this topic. We also tried to reduce bias by using two independent reviewers during the selection of articles and data extraction. However, the results of this study may have limited generalizability due to selection bias. This review was limited to five electronic databases (Medline, Ovid, PubMed, Science Direct and Scopus) and limited to articles published only in English. The search was only performed using online databases, and as such, unpublished articles were not considered. However, these databases have a very wide coverage of published medical journals including high quality and high impact journals.

In conclusion, various statistical methods have been used to measure agreement in validation studies. This study concludes that the Bland-Altman method is the most popular method that has been used to assess agreement between medical instruments measuring continuous variables. There were also some inappropriate applications of statistical methods to assess agreement found in recent medical literature. It is important for the clinician and medical researcher to be aware of this issue because erroneous and misleading conclusions from inappropriate statistical analyses may lead to the application of inaccurate instruments in clinical practice. The issue of inappropriate analyses in agreement studies needs to be highlighted to prevent repetition of the same mistake by future researchers.

## Supporting Information

Checklist S1 (PDF)

## References

[1] De Vet HCW, Armitage P, Colton T (1998). Observer reliability and agreement.. Encyclopedia biostatistica: John Wiley & Sons, Ltd Chicester.

[2] Altman DG, Bland JM (1983). Measurement in Medicine: the Analysis of Method Comparison Studies.. The Statistician.

[3] Luiz RR, Szklo M (2005). More than one statistical strategy to assess agreement of quantitative measurements may usefully be reported.. Journal of Clinical Epidemiology.

[4] Daly LE, Bourke GJ (2000). Interpretation and Use of Medical Statistics..

[5] Bland JM, Altman DG (1986). Statistical methods for assessing agreement between two methods of clinical measurement..

[6] Satia J, Galanko J (2007). Comparison of Three Methods of Measuring Dietary Fat Consumption by African-American Adults.. Journal of the American Dietetic Association.

[7] Reis M, Aniceto P, Aguiar P, Simao F, Segurado S (2007). Quantification of urinary chorionic gonadotropin in spontaneous abortion of pre-clinically recognized pregnancy: method development and analytical validation.. Int J Hyg Environ Health.

[8] Ten Boekel E, Böck M, Vrielink G-J, Liem R, Hendriks H (2007). Detection of shortened activated partial thromboplastin times: An evaluation of different commercial reagents.. Thrombosis Research.

[9] Allen R, Wallace L, Larson T, Sheppard L, Liu L-JS (2007). Evaluation of the recursive model approach for estimating particulate matter infiltration efficiencies using continuous light scattering data.. Journal Of Exposure Science & Environmental Epidemiology.

[10] Camara O, Schnabel JA, Ridgway GR, Crum WR, Douiri A (2008). Accuracy assessment of global and local atrophy measurement techniques with realistic simulated longitudinal Alzheimer’s disease images.. NeuroImage.

[11] Chovel Cuervo M, Sterling A, Abreu Nicot I, García Rodríguez M, Rodríguez García O (2008). Validation of a new alternative for determining in vitro potency in vaccines containing Hepatitis B from two different manufacturers.. Biologicals.

[12] Hof I, Arbab-Zadeh A, Dong J, Scherr D, Chilukuri K (2008). Validation of a Simplified Method to Determine Left Atrial Volume by Computed Tomography in Patients With Atrial Fibrillation.. The American Journal of Cardiology.

[13] Jaffrin MY, Morel H (2008). Body fluid volumes measurements by impedance: A review of bioimpedance spectroscopy (BIS) and bioimpedance analysis (BIA) methods.. Medical Engineering & Physics.

[14] Mündermann A, Dyrby C, Andriacchi T (2008). A comparison of measuring mechanical axis alignment using three-dimensional position capture with skin markers and radiographic measurements in patients with bilateral medial compartment knee osteoarthritis.. The Knee.

[15] Shuaibi A, Sevenhuysen G, House J (2008). Validation of a Food Choice Map with a 3-Day Food Record and Serum Values to Assess Folate and Vitamin B-12 Intake in College-Aged Women.. Journal of the American Dietetic Association.

[16] Ahn Y, Garruto RM (2008). Estimations of body surface area in newborns.. Acta Paediatrica (Oslo, Norway: 1992).

[17] Andrieux P, Kilinc T, Perrin C, Campos-Gimenez E (2008). Simultaneous determination of free carnitine and total choline by liquid chromatography/mass spectrometry in infant formula and health-care products: single-laboratory validation.. Journal Of AOAC International.

[18] Anderst W, Zauel R, Bishop J, Demps E, Tashman S (2009). Validation of three-dimensional model-based tibio-femoral tracking during running.. Medical Engineering & Physics.

[19] Barthelemy J, Gregor L, Krejci I, Wataha J, Bouillaguet S (2009). Accuracy of electronic apex locater-controlled handpieces.. Oral Surgery, Oral Medicine, Oral Pathology, Oral Radiology, and Endodontology.

[20] Di Noia J, Contento IR (2009). Use of a Brief Food Frequency Questionnaire for Estimating Daily Number of Servings of Fruits and Vegetables in a Minority Adolescent Population.. Journal of the American Dietetic Association.

[21] Hacihaliloglu I, Abugharbieh R, Hodgson AJ, Rohling RN (2009). Bone Surface Localization in Ultrasound Using Image Phase-Based Features.. Ultrasound in Medicine & Biology.

[22] Naidu S, Panchik D, Chinchilli V (2009). Development and Validation of the Hand Assessment Tool.. Journal of Hand Therapy.

[23] Satia J, Watters J, Galanko J (2009). Validation of an Antioxidant Nutrient Questionnaire in Whites and African Americans.. Journal of the American Dietetic Association.

[24] Cuker A, Ptashkin B, Konkle BA, Pipe SW, Whinna HC (2009). Interlaboratory agreement in the monitoring of unfractionated heparin using the anti-factor Xa-correlated activated partial thromboplastin time.. J Thromb Haemost.

[25] Ahn Y, Kwon E, Shim JE, Park MK, Joo Y (2007). Validation and reproducibility of food frequency questionnaire for Korean genome epidemiologic study.. European Journal Of Clinical Nutrition.

[26] Wikipedia The Free Encyclopedia: Bland-Altman plot.. http://en.wikipedia.org/wiki/Bland%E2%80%93Altman_plot.

[27] Hopkins WG (2004). Bias in Bland-Altman but not Regression Validity Analyses.. Sportscience.

[28] Ludbrook J (2002). Statistical techniques for comparing measurers and methods of measurement: a critical review.. Clin Exp Pharmacol Physiol.

[29] Davis JA, Rozenek R, Decicco DM, Carizzi MT, Pham PH (2007). Comparison of three methods for detection of the lactate threshold.. Clin Physiol Funct Imaging.

[30] Lee J, Koh D, Ong CN (1989). Statistical evaluation of agreement between two methods for measuring a quantitative variable.. Comput Biol Med.

[31] Bland JM, Altman DG (1990). A note on the use of the intraclass correlation coefficient in the evaluation of agreement between two methods of measurement.. Comput Biol Med.

[32] Fay MP (2005). Random marginal agreement coefficients: rethinking the adjustment for chance when measuring agreement.. Biostatistics.

[33] Bruton A, Conway JH, Holgate ST (2000). Reliability: What is it, and how is it measured?. Physiotherapy.

[34] Essack Y, Hoffman M, Rensburg M, Van Wyk J, Meyer CS (2009). A comparison of five glucometers in South Africa.. JEMDSA.

[35] Kottner J, Audigé L, Brorson S, Donner A, Gajewski BJ (2011). Guidelines for Reporting Reliability and Agreement Studies (GRRAS) were proposed.. Journal of Clinical Epidemiology.

[36] Hanbazaza SM, Mansoor I (2012). Accuracy evaluation of point-of-care glucose analyzers in the Saudi market.. Saudi Medical Journal.

